# Use of Neuroimaging in NHS Borders Memory Assessment Clinic: An Audit 10 Years on

**DOI:** 10.1192/bjo.2026.11849

**Published:** 2026-06-30

**Authors:** Calum Stewart, Lucy Calvert

**Affiliations:** 1 South East Deanery, Edinburgh, United Kingdom; 2 NHS Borders, Melrose, United Kingdom

## Abstract

**Aims:**

This audit aimed to assess current practice within NHS Borders Mental Health for Older Adults Service (MHOAS) regarding the use of neuroimaging in memory assessment clinics. Specifically, it sought to determine the proportion of patients with suspected dementia who had a documented decision and rationale regarding neuroimaging, and the proportion who underwent neuroimaging as part of their assessment. This was compared with findings from a previous audit conducted in 2014.

**Methods:**

Patients referred to NHS Borders Older Adult teams with suspected dementia between January and March 2024 were identified from administrative referral records. A retrospective review of initial cognitive assessment letters was undertaken between February and August 2025. Data collected included whether there was documented consideration and rationale for neuroimaging, and whether neuroimaging was subsequently performed. The agreed audit standard was that all patients with suspected dementia should have a documented decision and rationale regarding neuroimaging, and that the majority should undergo imaging unless a clear clinical justification was recorded.

**Results:**

Fifty-one patients were included in the audit. Documented consideration of neuroimaging was present in 45 patients (88.2%), representing a substantial improvement compared with 59% in the 2014 audit. Neuroimaging was performed in 27 patients (53%), compared with 18% in 2014. Of the 24 patients who did not undergo neuroimaging, eight (33%) had already received neuroimaging within the preceding two years, and repeat imaging was deemed clinically unnecessary. In the remaining cases, the most common documented rationale for not performing neuroimaging was that the diagnosis was already clear and that imaging was unlikely to add diagnostic value or alter management.

**Conclusion:**

This audit demonstrates a marked improvement over the past decade in both the documentation and utilisation of neuroimaging within the NHS Borders memory assessment clinic. Current practice is more closely aligned with national NICE and SIGN guidance, with appropriate clinical reasoning documented where neuroimaging is not performed. The findings suggest that neuroimaging is being used proportionately and appropriately, balancing guideline recommendations with principles of Realistic Medicine.

